# Potential of Landfill Mined Combustible Polymer Composite and Soil-like Fraction for Energy Recovery, Chemical Recycling, and Resource Recovery †

**DOI:** 10.3390/polym17182514

**Published:** 2025-09-17

**Authors:** Suyoung Lee, Tae Uk Han

**Affiliations:** 1 Environmental Resources Research Department, National Institute of Environmental Research (NIER), Hwangyeong-ro 42, Incheon 22689, Republic of Korea; ssyy76@korea.kr; 2 Department of Graduate School of Convergence Science, Environmental Energy Engineering, Seoul National University of Science and Technology, Gongneung-ro 232, Seoul 01811, Republic of Korea

**Keywords:** landfill mining, energy recovery, chemical recycling, resource recovery, circular economy

## Abstract

The landfill mining and reclamation (LFMR) project is increasingly recognized as crucial for achieving sustainable waste management and supporting global environmental goals, such as the United Nations Sustainable Development Goals related to clean energy, responsible consumption, and sustainable cities. This study evaluated the potential of combustible polymer composites (CPCs) derived from landfill mining waste for energy recovery and chemical recycling as well as resource recovery potential of soil-like fractions (SLFs). Through physico-chemical analysis and pyrolysis reaction with catalytic upgrading process, the study evaluates the suitability of CPCs for energy recovery as a solid recovered fuel (SRF) and chemical recycling feedstock. For assessing the SLFs for potential use as recycled aggregates and cover materials, total organic carbon, heavy metal concentration, and biodegradability were investigated. CPCs exhibited varied SRF and chemical feedstock qualities depending on site-specific polymer composition, while SLFs met environmental criteria for both inert waste and stabilization soil classification. The findings not only highlight technical feasibility, but also provide a transferable evaluation framework supporting ‘circular economy’ policies. Therefore, LFMR projects can contribute to sustainable waste management and energy production and provide solutions for effective material recycling, aligning with global environmental and resource conservation goals.

## 1. Introduction

With the strengthening of waste reduction and energy and/or resource recovery policies, initiatives are underway to reduce landfill disposal and enhance the recovery of resources from existing landfill waste globally. One such initiative is the landfill mining and reclamation (LFMR) project [1,2,3,4]. This approach enhances the usability of existing landfill sites, contributing to regional development while providing advantages of substituting fossil fuels and reducing greenhouse gas emissions through energy recovery from mined landfill waste [4]. In South Korea, the LFMR project has been ongoing since 2009 as part of the “Zero Waste to Landfill” project. Additionally, the Ministry of Environment in South Korea has enacted legislation under the Waste Management Act that prohibits the direct landfill disposal of municipal solid waste without intermediate treatment (sorting, recycling, and incineration), starting in 2026 [5].

South Korea imports 90% and 97% of its resources and energy, respectively, owing to its limited natural resources and low energy self-sufficiency. Therefore, resource and energy recovery from mined landfill waste considerably contribute to sustainable development and waste management [6]. Mined landfill waste generated through LFMR projects is typically classified into combustible polymer composites (CPCs) and soil-like fractions (SLFs) [1]. CPCs are primarily composed of plastics, and their utilization can be categorized into three main options: (1) direct reuse without modification (mechanical recycling), (2) production of solid recovered fuel (SRF) followed by incineration to recover thermal energy [4], and (3) the use of CPCs as feedstock for chemical recycling (pyrolysis) to produce petrochemicals [2,3,7]. Direct reuse is often considered less feasible owing to the mixed composition of CPCs, which typically contain various plastics and other polymers [1]. Therefore, other two options can be promising strategies for CPCs utilization.

In addition, SLFs can be reused as daily soil cover and recycled aggregates [1,8,9]. The most critical factor in SLF recycling is soil stability, which can be assessed by measuring the total organic carbon (TOC) content, heavy metal concentration, and biodegradability (aerobic respiration activity). According to the EU Landfill Directive, waste is categorized into three types: inert, non-hazardous, and hazardous [10,11]. In particular, waste with a TOC < 3 wt. % and heavy metal concentrations below the limits specified in Table S1 (Supplementary Information) are classified as inert waste. South Korea defines stabilized landfill soil as having a biodegradability of <5 mg O_2_/g based on the guidelines for maintenance and post-management of landfill sites. Therefore, to use SLFs as daily soil cover or recycled aggregates, they must meet three criteria: TOC < 3 wt. %, heavy metal concentrations within inert waste limits, and biological stabilization <5 mg O_2_/g.

Although numerous studies have investigated the valorization of landfill-mined waste, most existing literature focuses on either CPCs or SLFs separately. For instance, the feasibility of producing SRF from landfill plastics and co-pelletizing with organic sludge has been reported [12], the influence of landfill aging on pyrolysis characteristics has also been explored [13], and the incineration and gasification routes were compared for energy recovery from mined CPCs [14]. Simultaneously, research into SLFs has focused on their potential as fill material [8], road subbase material [15], and their geotechnical properties for reuse in infrastructure [16]. These studies have contributed to advancing recovery technologies for specific waste components. However, they rarely assess CPCs and SLFs together under an integrated framework, and do not simultaneously evaluate their energy recovery, chemical recycling, and material reuse potential from a holistic perspective. Therefore, there remains a critical need for comprehensive research addressing the combined recovery potential of both CPCs and SLFs, particularly in the context of ‘Circular Economy’ strategies. This study aims to fill this research gap by simultaneously analyzing the energy recovery, chemical recyclability, and resource potential of CPCs and SLFs obtained from landfill mining sites in South Korea.

This study is significant as it provides an integrated assessment framework for both CPCs and SLFs recovered through LFMR. Unlike prior studies that focused on single waste streams, our work demonstrates how simultaneous recovery strategies can support national resource independence and align with global sustainability goals (e.g., the United Nations Sustainable Development Goals for clean energy, responsible consumption, and sustainable cities). This study is guided by the following research questions: (1) How can CPCs from LFMR be effectively evaluated for both energy and chemical recovery potential? (2) Can SLFs meet the standards for safe reuse in civil and environmental applications? (3) What framework can be proposed to assess the dual-stream resource recovery from LFMR?

Therefore, in this study, we evaluated the energy recovery, chemical recycling, and resource recovery potential of CPCs and SLFs from landfill mining waste by comparing and analyzing their physicochemical, pyrolysis, and soil stability characteristics. Four types of CPCs and SLFs were used in this study. The energy recovery potential of CPCs as SRF feedstock was assessed based on their physicochemical characteristics, including the lower heating value (LHV), water and ash content, and chlorine concentration (Cl wt. %) [17]. LHV, water and ash content are economic parameters of SRF. LHV represents the total energy content of waste for heat production, whereas water and ash content affect the energy cost savings of SRF-utilizing facilities. The Cl concentration is an environmental and facility-related factor. High Cl concentrations in combustible waste can lead to corrosion, slagging, and fouling in boilers and facilities and are associated with the production of dioxins/furans, which are air pollutants [17]. Pyrolysis-gas chromatography/mass spectrometry (Py-GC/MS) analysis was performed to evaluate the chemical recycling potential of CPCs. Additionally, catalytic pyrolysis of CPCs over HZSM-5(30) acidic catalyst was conducted to investigate the conversion efficiency of the pyrolytic vapor produced from CPCs pyrolysis into BTEXs (benzene, toluene, ethylbenzene, and xylenes) and styrene, which can be used as gasoline or petrochemical feedstock [18]. Finally, the TOC content, heavy metal content, and biodegradability of the SLFs were analyzed to elucidate the resource recovery potential of each SLFs.

## 2. Materials and Methods

### 2.1. Landfill Sites and Sample Preparation

Table 1 presents the characteristics of the four landfill sites, including the type, capacity, and location, for LFMR purposes. Mined waste (300–500 kg) was collected from each landfill site. The waste was screened and separated using the trommel screen method to prepare the representative mined landfill waste sample. Subsequently, the mined landfill waste was further classified into the CPCs and SLFs through manual separation. Figure S1 in the supplementary information shows the CPCs and SLFs of the mined landfill waste. Notably, mined landfill waste of site A had the lowest CPCs portion (the highest SLFs portion) because of its landfill type. The landfill type of site A was classified as an unsanitary landfill, indicating no distinction existed between landfill waste and cover materials, such as liners and soil [19]. The separated CPCs were milled and sieved to particle sizes <2 mm using a cryo-ball mill with liquid nitrogen to evaluate the energy recovery and chemical recycling potential. In addition, SLFs with particle sizes of <100 mm were also prepared and used as a sample for evaluating the resource recovery potential.

### 2.2. Evaluation of Energy Recovery Potential

To evaluate the energy recovery potential of CPCs (the potential for SRF production), physicochemical properties such as proximate analysis, ultimate analysis, and LHV measurements were performed. The proximate analysis was conducted according to the Korean Standard Test Method for Waste (ES 06000.b). For ultimate analysis, an elemental analyzer (628 series, LECO Co., St. Joseph, MI, USA) was used to quantify carbon (C), hydrogen (H), nitrogen (N), and sulfur (S) concentrations. In addition, to calculate the LHV of each CPC, the higher heating value was first measured using a Leco AC500 bomb calorimetry, and the LHV was subsequently determined using Dulong’s formula [20]. The Cl concentration was also analyzed using the combustion-ion chromatography method in accordance with the SRF quality test and analysis methods in Korea [6]. CPCs (1 g) was placed in an oxygen bomb containing 10 mL of distilled water. Compressed oxygen was gradually introduced into the bomb until the pressure reached 3.0 to 3.5 MPa. After the combustion process, the bomb was allowed to cool to room temperature for at least 10 min. The sampling solution prepared for this procedure was analyzed using ion chromatography (940 Professional IC Vario, Metrohm Co., Herisau, Switzerland).

In addition, the unit energy cost (USD/kWh) was calculated based on the LHV of CPCs and the total operating cost for evaluating the economic feasibility for energy recovery. The LHV of CPCs was assumed to be 35 MJ/kg (i.e., average value of four CPCs was about 35 MJ/kg), resulting in a total energy output of 35,000 MJ/ton. This energy output (MJ) was then converted to kWh using the conversion factor (1 kWh = 3.6 MJ), yielding approximately 9722 kWh/ton. The unit energy cost was calculated by dividing the total cost including SRF production and facility operational costs (USD/ton) by the total energy output (kWh/ton).

### 2.3. Evaluation of Chemical Recycling Potential

To investigate the chemical recycling potential, the pyrolytic compounds produced in a micro type pyrolyzer (EGA/Py-3030D, Frontier Laboratories Ltd., Koriyama, Japan) were analyzed using GC/MS (Agilent 7890A/5975C inert, Santa Clara, CA, USA). A 1.0 mg sample of CPCs and 4.0 mg of the catalyst/CPC mixture (with a 3:1 catalyst-to-CPCs ratio) were prepared in a deactivated stainless steel sample cup for the thermal and catalytic pyrolysis reaction, respectively. For the catalytic pyrolysis reaction, calcinated microporous acidic HZSM-5(30) catalyst, purchased from Zeolyst, was used. The properties of the HZSM-5 catalyst are presented in the Supplementary Information (Figure S2 and Table S2). Then, each prepared sample was dropped on a preheated pyrolyzer at 600 °C, and the pyrolytic compounds produced by the pyrolyzer were moved to a capillary metal column (UA-5, 30 m × 0.25 mm × 0.25 μm) connected to a GC inlet (320 °C, 100:1 split ratio). The pyrolytic vapors mixture was cryo-focused using liquid nitrogen (−195 °C, 2 min) and separated according to programmed oven temperature (40 °C maintained for 5 min → increased oven temperature at 10 °C/min → 320 °C maintained for 5 min). The separated compounds were detected using a single quadrupole MS (scan range: *m*/*z* 18–600), and each peak in the chromatogram was identified using two types of MS libraries (NIST 8th, Agilent Technologies and F-Search, Frontier Laboratories Ltd.). To evaluate the amount of each compound formed, the absolute MS peak areas were integrated and compared with respect to the CPCs type, and the average value of three replicates was used to confirm the reproducibility of the data.

### 2.4. Evaluation of Resource Recovery Potential

To assess the feasibility and safety of using SLFs as landfill cover materials and other recycled aggregates, TOC, aerobic respiration activity (AT_4_), and heavy metal leaching characteristics of the SLF were investigated. The TOC content was determined as the difference between total carbon and total inorganic carbon using a TOC analyzer (Multi EA 4000, Analytikjena Co., Jena, Germany). The AT_4_ test was conducted using an Oxitop Controller OC110 following established procedures [21]. Although an AT_4_ value below 5.0 mg O_2_/g for 4 days indicates biologically stable landfill waste according to the German ordinance, we conducted the test for 10 days to further monitor the respiration behavior of aerobic microbes. Additionally, the heavy metal leaching characteristics of SLFs were analyzed using inductively coupled plasma-optical emission spectroscopy (ICP-OES, Optima 8300, PerkinElmer Co., Shelton, CT, USA) to measure the heavy metal concentrations specified by the EU Landfill Directive [11].

## 3. Results and Discussion

### 3.1. Energy Recovery Potential of Polymer Fraction

**Figure 1:**
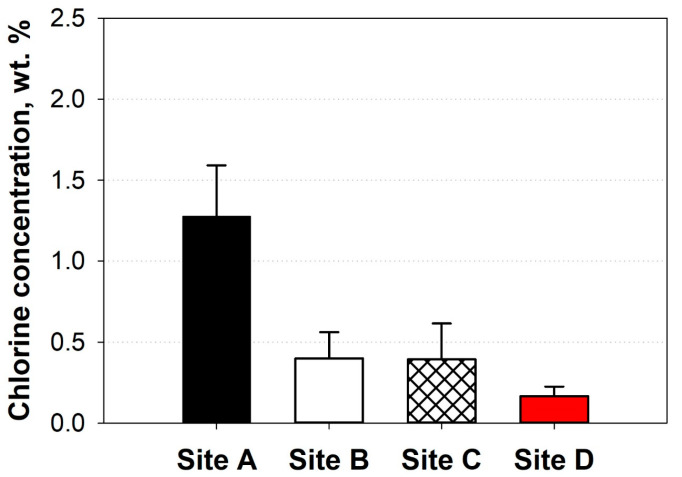
The chlorine concentrations of CPCs.

Table 2 presents the physicochemical properties of the CPCs. The water content of each CPC ranged from 5.72 to 36.10 wt. %, indicating that the direct use of CPCs as SRF, except for CPCs-Site D, was limited because the water content did not meet the SRF quality standard of Korea (≤10 wt. %) [6]. Although the EU does not regulate the water content in SRF (determining the content by the customer) [22], the water content of CPCs as raw materials for SRF should be minimized by a drying process because the efficiency of heat and electricity production (boiler efficiency) decreases as the water content in SRF increases [17,23]. Meanwhile, the ash content and LHV of CPCs were in the acceptable range in the SRF quality standard of Korea (ash ≤ 20 wt. % and LHV ≥ 14.6 MJ/kg) [6]. This indicates that each CPCs may be composed of various types of plastics, such as polyethylene (PE), polypropylene (PP), and polystyrene (PS), because the synthesis polymer has a higher heating value > 40 MJ/kg and a lower content of water and ash < 1.0 wt. % [24,25,26]. However, the ultimate analysis results indicated that CPCs are not solely composed of hydrocarbon-based plastics because they do not include heteroatoms such as oxygen, nitrogen, sulfur, and Cl in their chemical structures. This indicates that the CPCs could be composed of hydrocarbon-based plastics as well as heteroatom-containing plastics, such as polyethylene terephthalate (PET), polyvinyl chloride (PVC), and polymethyl methacrylate (PMMA), together with biomass. Lignocellulosic biomass consists of cellulose, hemicellulose, and lignin, which contain large amounts of oxygen in their structure [27]. To further understand the suitability of CPCs as SRF feedstock, we investigated the Cl concentration in the combustion gas (Figure 1), which generates hydrogen chloride (HCl) and leads to the corrosion of thermal energy facilities [28]. The Cl concentrations of all the CPCs were lower than 2.0 wt. %, complying with the SRF quality standards in South Korea. In addition, the Cl concentration in the CPCs was lower than the EU standard value [6]. In the EU, the Cl concentration in the SRF is regulated to be below 3.0 wt. %. Consequently, this suggests that the use of all CPCs as SRF feedstock is environmentally feasible.

To assess the energy recovery potential of each CPCs as a raw material for SRF production, four parameters (LHV, water content, ash content, and Cl concentration) were evaluated according to the score criteria for each parameter, which were developed to evaluate the energy recovery potential in this study (Table 3). The CPCs-Site D had the highest potential to serve as a superior-grade SRF feedstock (point = 16). It can be explained due to the higher scores at water and ash contents at CPCs-Site D compared to other CPCs. Therefore, it means that CPCs can be a high-quality feedstock for manufacturing SRF through pretreatment techniques such as drying for water removal and leaching for ash removal by water or chemicals [29]. Although upgrading the SRF class can be achieved through pretreatment methods, evaluating the economic feasibility of these methods is important because they require external energy for drying, and generate large amounts of secondary wastewaters for ash removal.

From an economic perspective, the cost analysis reveals that even when including an operational cost of 80 USD/ton [30], CPCs-derived SRF has the potential to be competitive compared to conventional fossil fuels. For example, assuming a low SRF production cost scenario of 6 USD/ton [31], the unit energy cost is approximately 0.0089 USD/kWh. Meanwhile, the cost of a higher coast scenario of 60 USD/ton [32] results in 0.014 USD/kWh. Both values are significantly below the unit energy coast of coal-fired power generation in South Korea (0.06~0.07 USD/kWh reported by Korea Electric Power Corporation in 2022). In addition, a sensitivity analysis further shows that CPCs-derived SRF lose its economic advantage over coal when the costs combined production SRF and operation facility exceed approximately 580–680 USD/ton. Therefore, it means that the conversion of CPCs into SRF ensures economic viability in addition to its environmental benefits.

### 3.2. Chemical Recycling Potential of Polymer Fraction

**Figure 2:**
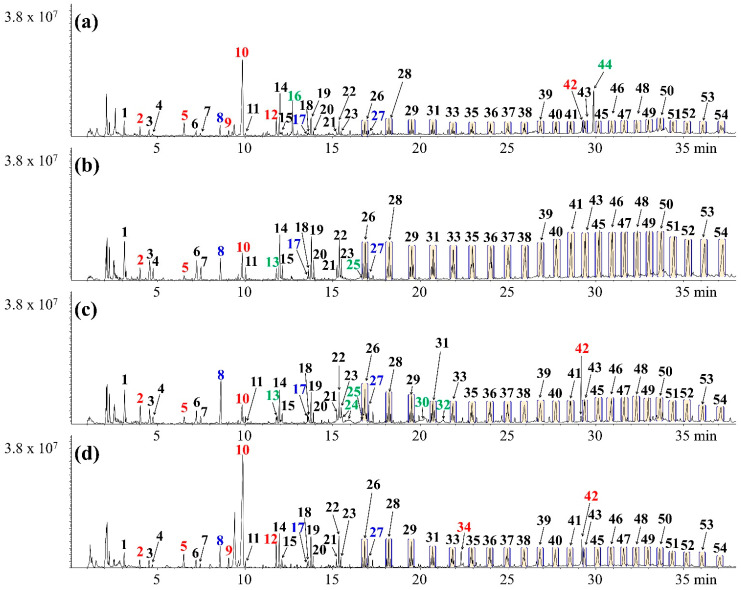
Chromatograms obtained from the non-catalytic pyrolysis of (**a**) CPCs-Site A, (**b**) CPCs-Site B, (**c**) CPCs-Site C, and (**d**) CPCs-Site D.

Figure 2 shows the non-catalytic pyrograms of the CPCs pyrolyzates, and the peak areas of the CPCs pyrolysis products are listed in Table S3. Many different types of compounds, such as aliphatic hydrocarbons, aromatics, acids, and oxygenates, were produced because of the different compositional properties of each CPCs. Large amounts of specific pyrolyzates of HDPE (alkadienes, alkenes, and alkanes) [33,34] were detected in all CPCs chromatograms. Thus, HDPE was confirmed to be the major component of all CPCs. In addition, alkylated hydrocarbons such as 2,4-dimethyl-1-heptene (# 8), 2,4,6-trimethyl-1-nonene (# 17), and 2,4,6,8-tetramethyl-1-undecene (# 27) were simultaneously produced in all CPCs pyrolysis reactions. These are typical PP pyrolyzates [35], it indicates that PP was major component of CPCs together with HDPE. Notably, the peak area of 2,4-dimethyl-1-heptene (# 8) at CPCs-Site C was larger than that of the other CPCs, which means that the amount of PP at CPCs-Site C was higher than that at the other sites.

Although styrene (#10), a specific pyrolyzate of PS [36], was also produced along with aliphatic hydrocarbons (pyrolyzates of HDPE and PP), a significant difference was observed in the peak area of each CPCs. The peak area of styrene was in the order CPCs-Site D (210.9 × 10^7^) > CPCs-Site A (109.58 × 10^7^) > CPCs-Site B (28.19 × 10^7^) > CPCs-Site C (19.26 × 10^7^), indicating that the PS fraction of CPCs was highest at CPC-Site D. Notably, kinematic viscosity at 40 °C of PS pyrolysis oil (1.03 mm^2^ s^−1^) is lower than that of HDPE and PP pyrolysis oil (1.70 mm^2^ s^−1^ for HDPE and 1.60 mm^2^ s^−1^ for PP) owing to the presence of aromatic structure of styrene [37]. The high kinematic viscosity of oil suggests that the combustion efficiency between fuel and air can be reduced owing to problems inside a pipe with fluid flow [38]. In addition, the CPCs-Sites A, B, and C, except for CPCs-Site D, included other polymers such as PET (#24 benzoic acid, #32 terephthalic acid, and #44 bis(2-ethylhexyl) phthalate, which are major pyrolyzates of PET [39]), and wood (#30 levoglucosan, which is a typical pyrolyzate of lignocellulosic biomass [40]). This indicates that the oil produced by pyrolysis at CPCs-Site D (styrene-rich and low-oxygen pyrolysis oil) can be advantageous as a fuel or additive to increase fuel quality.

Although the oil produced from the pyrolysis reaction of CPCs has the potential to be used as a fuel, the commercialization of oil as a fuel and chemical feedstock can be limited owing to its proportion. Large-molecule hydrocarbons (waxy hydrocarbons) can cause oil condensation and block pipelines owing to their high viscosity [37,38,41]. Therefore, catalytic pyrolysis of CPCs over HZSM-5(30) was performed to investigate the production of value-added chemical products (aromatic hydrocarbons).

**Figure 3:**
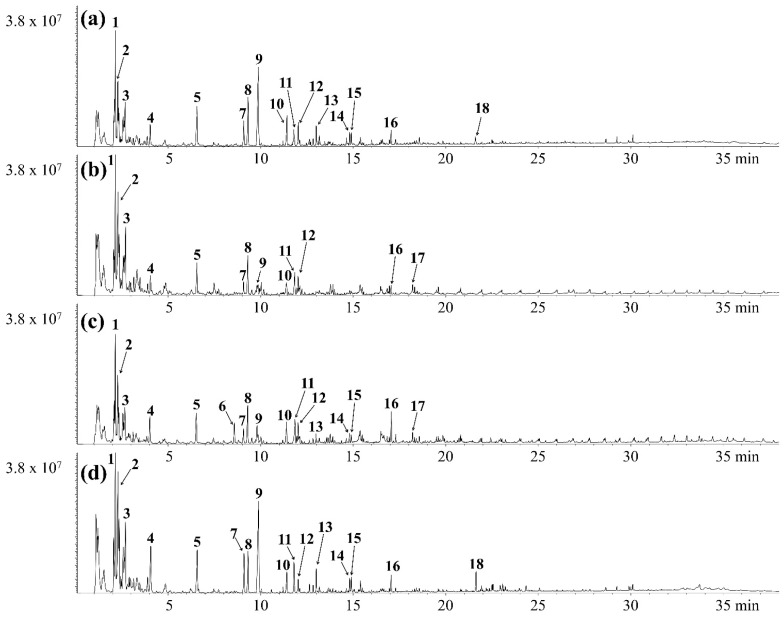
Chromatograms obtained from the catalytic pyrolysis of (**a**) CPCs-Site A, (**b**) CPCs-Site B, (**c**) CPCs-Site C, (**d**) CPCs-Site D over HZMS-5(30).

Figure 3 and Table 4 show the chromatograms and product peak areas for the catalytic pyrolysis of CPCs over HZSM-5(30). Compared to the non-catalytic pyrolysis of CPCs, the amounts of aliphatic hydrocarbons, which are specific pyrolyzates of HDEP and PP, were notably reduced. This is because waxy hydrocarbons are converted into high-value-added aromatics such as benzene, toluene, ethylbenzene, and xylene (BTEXs) through catalytic reactions such as isomerization, oligomerization, cyclization, and aromatization [42]. In particular, HZSM-5 is a well-known “shape-selective catalyst,” indicating that it has a favorable structure (the MFI structure) for converting aliphatic hydrocarbons into BETXs [43]. As a result, the pyrolysis oil obtained from catalytic pyrolysis of CPCs significant amounts of BTEXs together with styrene. These compounds are fundamental petrochemical intermediates in the industry with broad applications [44]. Benzene (#4) is a critical precursor for styrene, phenol and nylon, serving as a major feedstock for resins and synthetic fibers. In case of toluene (#5), it is widely used as an industrial solvent, and also functions as a high-octane additive in gasoline blending. Ethylbenzene (#7) is used as solvent in inks and dyes. Xylene (#8) is used as a key intermediate for polyester fibers and PET bottles. In addition, it should be noted that styrene (#9) is one of the most important monomers for the plastic (PS and acrylonitrile-butadiene-styrene resin) industry. Therefore, BTEXs and styrene rich oil can serve as valuable feedstocks for both fuel blending and petrochemical manufacturing. In terms of monoaromatic hydrocarbon formation (#4, 5, and 7–12), the potential of CPCs-Site D for value-added compound production was the highest, followed by CPCs-Sites A, C, and B. These results indicate that the CPCs-Site D have the highest potential for fuel and/or chemical feedstock production.

### 3.3. Resource Recovery Potential of Soil-like Fraction

**Figure 4:**
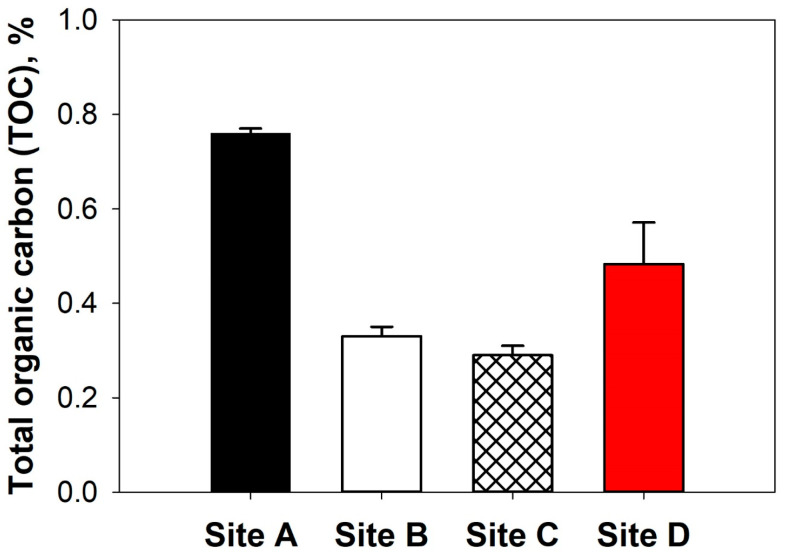
TOC concentrations of SLFs.

To evaluate the resource recovery potential of SLFs, the TOC and heavy metal concentrations in the SLF were investigated. The EU Landfill Directive established limit values for TOC and heavy metals regarding categorized landfill waste (inert, non-hazardous, and hazardous waste) [10,11]. In particular, inert waste designated for landfilled at specific sites must maintain a TOC level of <3.0 wt. % to prevent mixing with other unstable wastes. Therefore, when the TOC value is <3.0 wt. %, the SLF can be considered as inert and/or a stabilization material. Figure 4 shows the TOC concentration of the SLF, and the results revealed that the values of all the SLFs were <1.0 wt. %.

**Figure 5:**
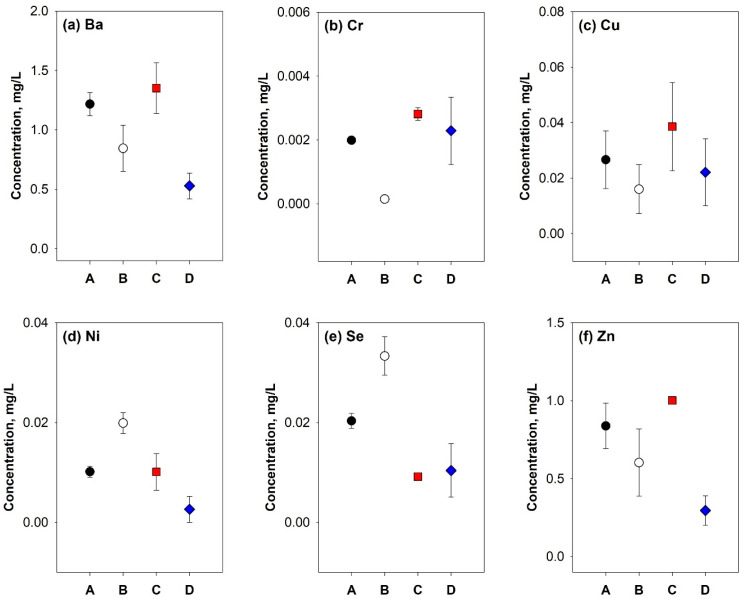
Heavy metal concentrations of SLFs determined using leaching percolation test.

Together with the TOC concentration, the elution characteristics of heavy metals in SLFs are also important when evaluating resource potential. Elute criteria of heavy metal concentration for inert waste in EU are As < 0.06 mg/L, Ba < 4.0 mg/L, Cd < 0.02 mg/L, Cr < 0.1 mg/L, Cu < 0.6 mg/L, Mo < 0.2 mg/L, Ni < 0.12 mg/L, Pb < 0.15 mg/L, Sb < 0.1 mg/L, Se < 0.04 mg/L, and Zn < 1.2 mg/L (Table S1). Among these, As, Cd, Mo, Pb, and Sb were not detected in the elution tests of the SLFs, and the concentrations of other heavy metals were below the EU standards for inert waste (Figure 5). This confirms that all SFLs have a physicochemical stability that is suitable for recycling materials.

**Figure 6:**
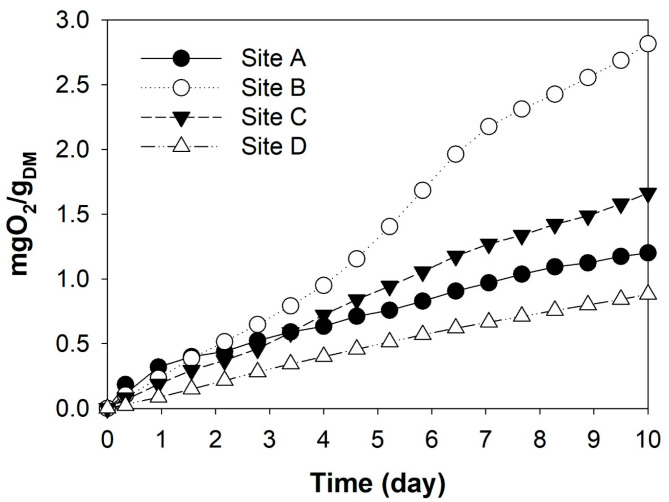
Time profiles of oxygen consumption of SLFs.

To further confirm the potential of resource recycling, the biodegradability of SLFs was also investigated based on the AT_4_ test results (an AT_4_ value of <5.0 mg O_2_/g for 4 days is considered biologically stable landfill waste according to the German ordinance). Figure 6 shows the time profiles of oxygen consumption of the SLFs and reveals that the values of all SLFs were <5.0 mg O_2_/g. Therefore, SLFs are biologically stable and can be recycled as material resources for covering and embankment purposes.

## 4. Challenges and Perspectives

This study is based on the circular economy framework, emphasizing material loops, waste valorization, and reduced dependency on virgin resources. By applying circular economy principles to landfill mining, we explore practical approaches to re-introduce excavated waste materials into industrial and municipal use cycles. Successfully implementing LFMR projects for energy recovery with chemical recycling from CPCs, as well as resource recovery of SLFs, requires various tasks.

The first challenge for energy recovery and chemical recycling is the diverse polymer composition in CPCs. The study results indicated that CPCs comprise a mixture of various plastics, including HDPE, PP, PS, PET, and biomass together with other polymers. This suggests that an enhanced waste sorting/separation system is necessary to produce uniform and high-quality SRF and pyrolytic oil. Consequently, the Korean government is making considerable investments in developing waste sorting systems and expanding pyrolysis plants as chemical recycling facilities as part of its recycling promotion and circular economy policy [45]. The Seo-gu of Incheon has recently proposed establishing a public pyrolysis facility on land within the metropolitan landfill, specifically at the SUDOKWON Landfill Site Management Corporation. The facility is currently undergoing a feasibility assessment, with operations anticipated to start in 2027. In particular, the Corporation already has the facility to manufacture SRF for energy recovery. The fundamental data presented in this study will be crucial in evaluating the commercialization feasibility of energy recovery and chemical recycling of CPCs from the LFMR project, considering both environmental and economic factors.

Another concern is the contamination of soil and groundwater with heavy metals owing to the use of SLFs as daily cover material and recycled aggregates. Although the SLFs, which were used in this study, had lower levels of heavy metal concentrations than those of EU inert waste, the presence of heavy metals still presents a substantial environmental challenge. Therefore, the environmental assessment of recycling is necessary. In South Korea, the assessment system is already established and implemented under the Waste Management Act [46] to promote the recycling of waste while minimizing environmental impacts. Based on the findings of this study, which indicate that SLFs have resource recovery potential, further assessments should be conducted to evaluate the feasibility of using SLFs as actual recycling materials.

## 5. Conclusions

This study highlights the potential of the materials recovered from landfill mining for energy and resource recovery, as well as chemical recycling.

- CPCs revealed high LHV and acceptable Cl concentrations for energy recovery.
- Styrene-rich pyrolytic oils were produced by CPCs pyrolysis reaction.
- Catalytic pyrolysis of CPCs enhanced the yield of high-value BTEXs and styrene.
- SLFs showed chemical and biological stability for resource recovery.

There are also three practical and policy implications in study. (1) Industries can utilize the CPC pyrolysis profile to design pretreatment processes for optimized fuel and chemical feedstock recovery. (2) SLFs that meet inert waste standards can reduce landfill burden and be safely reused in embankments or landscaping. (3) Policy-makers can refer to this framework when drafting regulations related to post-landfill mining material reuse.

## Figures and Tables

**Table 1:** Characterization of landfill sites.

Site	Type	Capacity (m^3^)	Mining Purpose	Location
A	Unsanitary	357,192	Development of a residential complex	Incheon
B	Secure for industrial waste	2,562,000	Maintenance and redesign of the landfill site to enlarge landfill capacity	Gunsan
C	Sanitary	2,897,000		Mokpo
D	Sanitary	1,508,000		Gyeong-Ju

**Table 2:** Physicochemical properties of combustible polymer composites.

Site	Proximate Analysis (wt. %)			Ultimate Analysis (wt. %)					Heating Value (MJ/kg)
	Water	Volatiles	Ash	C	H	N	S	Others	
A	18.73	65.18	16.09	67.02	9.20	0.25	0.28	23.25	34.28
B	36.10	51.44	12.46	70.82	11.04	0.41	0.34	17.39	36.04
C	20.04	62.48	17.47	64.97	9.68	0.33	0.09	24.93	32.70
D	5.72	85.25	9.03	74.88	10.00	0.31	0.14	14.67	39.30

**Table 3:** Evaluation of energy recovery potential as solid recovered fuel (SRF).

Point	LHV (MJ/kg)	Water (wt. %)	Ash (wt. %)	Cl Concentration (wt. %)	SRF Classification (Sum of Points)
**5**	≥35.0	≤5.0	≤5.0	≤0.5	Class 1 ≥ 15 10 ≤ Class 2 ≤ 14 9 ≤ Class 3 ≤ 13 4 ≤ Class 4 ≤ 8 Class 5 ≤ 3
**3**	≥30.0	≤10.0	≤10.0	≤1.0	
**1**	≥25.0	≤25.0	≤20.0	≤2.0	
Landfill site	LHV (MJ/kg)	Water (wt. %)	Ash (wt. %)	Cl Concentration (wt. %)	Sum of points (Classification)
**A**	34.3	18.7	16.1	1.28	**6** **(Class 4)**
**Point**	**3**	**1**	**1**	**1**	
**B**	36.0	36.1	12.5	0.40	**11** **(Class 2)**
**Point**	**5**	**0**	**1**	**5**	
**C**	32.7	20.0	17.5	0.39	10 **(Class 2)**
**Point**	**3**	**1**	**1**	**5**	
**D**	39.3	5.7	9.0	0.17	**16** **(Class 1)**
**Point**	**5**	**3**	**3**	**5**	

**Table 4:** MS peak area of evolved chemicals under the catalytic pyrolysis reaction of CPCs.

Peak No.	Compound	MS Peak Area (×10^−7^)			
		CPCs-Site A	CPCs-Site B	CPCs-Site C	CPCs-Site D
1	Propylene	68.3 ± 7.6	116.6 ± 15.8	71.5 ± 6.6	111.8 ± 13.6
2	1-Butene	55.7 ± 8.0	115.1 ± 17.1	55.7 ± 8.6	88.1 ± 17.8
3	2-Methyl-1-butene	41.0 ± 7.1	71.7 ± 17.5	37.3 ± 7.4	49.9 ± 7.8
4	Benzene	19.9 ± 2.6	17.5 ± 0.7	32.6 ± 4.6	46.8 ± 4.6
5	Toluene	40.7 ± 0.9	32.2 ± 1.1	30.9 ± 0.8	43.3 ± 2.2
6	2,4-Dimethyl-1-heptene	-	-	14.0 ± 4.8	-
7	Ethylbenzene	19.5 ± 1.2	11.0 ± 0.7	14.5 ± 0.3	33.7 ± 0.7
8	Xylene	41.7 ± 1.9	36.9 ± 1.8	31.2 ± 2.5	33.7 ± 1.7
9	Styrene	90.6 ± 5.8	8.9 ± 0.0	14.0 ± 0.3	132.3 ± 8.3
10	Ethyltoluene	22.5 ± 0.7	17.2 ± 2.8	17.9 ± 1.3	15.5 ± 1.0
11	Cyanobenzene + Methylstyrene	14.1 ± 1.9	17.1 ± 1.9	30.7 ± 4.3	23.7 ± 0.4
12	Trimethylbenzene	15.5 ± 0.8	14.3 ± 0.6	14.3 ± 0.7	10.4 ± 0.3
13	Indene	11.3 ± 0.5	-	6.8 ± 0.2	14.8 ± 0.2
14	1-Methylindene	7.2 ± 0.4	-	6.7 ± 0.2	7.8 ± 0.6
15	3-Methylindene	8.2 ± 0.4	-	7.9 ± 1.1	11.2 ± 0.3
16	Methylnaphthalene	9.6 ± 1.0	8.3 ± 1.1	19.3 ± 2.3	12.1 ± 1.0
17	Diphenyl	-	10.0 ± 0.9	7.7 ± 0.4	-
18	1,3-Diphenylpropane	5.1 ± 0.9	-	-	11.1 ± 1.9

## Data Availability

The original contributions presented in the study are included in the article; further inquiries can be directed to the corresponding author.

## Supplementary Materials

The following supporting information can be downloaded at: https://www.mdpi.com/article/10.3390/polym17182514/s1. Table S1. EU landfill limit values for inert waste. Table S2. The N_2_ sorption results of HZSM-5(30) catalyst used in this study. Table S3. MS peak area of evolved chemicals under the pyrolysis reaction of CPCs. Figure S1. The CPCs and SLFs fraction (wt. %) of mined landfill waste. Figure S2. NH_3_-TPD curve of HZSM-5(30).
