# Supplementary material for: Potential of Landfill Mined Combustible Polymer Composite and Soil-like Fraction for Energy Recovery, Chemical Recycling, and Resource Recovery
Source: Polymers (Basel). 2025 Sep 17;17(18):2514. doi: 10.3390/polym17182514 (PMC12473329; doi:10.3390/polym17182514)
Supplement: Supplementary file 1 [file polymers-17-02514-s001.zip › polymers-3855014-supplementary.pdf]

polymers

MDPI

Supplementary Information

Potential of Landfill Mined Combustible Polymer Composite and Soil-like Fraction for Energy Recovery, Chemical Recycling, and Resource Recovery †

Suyoung Lee [1,2] and Tae Uk Han [1,*]

[1] Environmental Resources Research Department, National Institute of Environmental Research (NIER), Hwangyeong-ro 42, Incheon 22689, Republic of Korea; ssyy76@korea.kr
[2] Department of Graduate School of Convergence Science, Environmental Energy Engineering, Seoul National University of Science and Technology, Gongneung-ro 232, Seoul 01811, Republic of Korea
* Correspondence: taeukhan@korea.kr; Tel.: +82-32-560-7562
† Dedicated to Dr. Kyuyeon Kim on the occasion of his retirement from the National Institute of Environmental Research (NIER).

Section 1. Introduction

Table S1. EU landfill limit values for inert waste.

Index	TOC (wt. %)	Heavy metal (mg/L)											
		As	Ba	Cd	Cr	Cu	Hg	Mo	Ni	Pb	Sb	Se	Zn
Conc.	3.0	0.06	4	0.02	0.1	0.6	0.002	0.2	0.12	0.15	0.1	0.04	1.2

Section 2. Materials and Methods

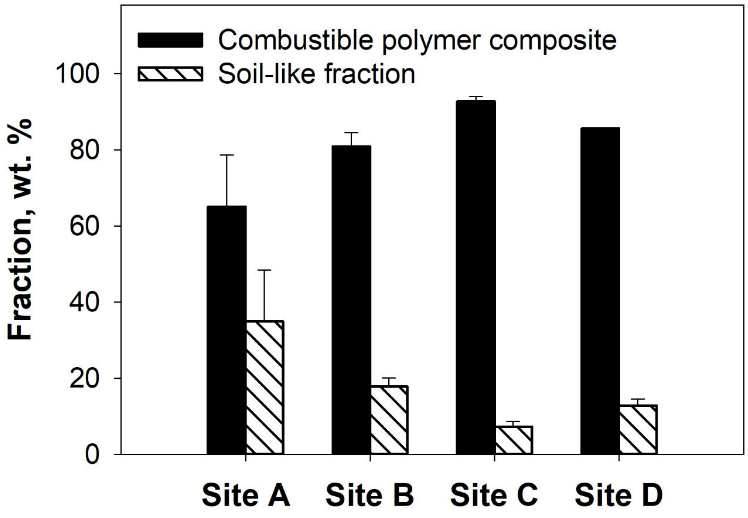

Figure S1. The CPCs and SLFs fraction (wt. %) of mined landfill waste.

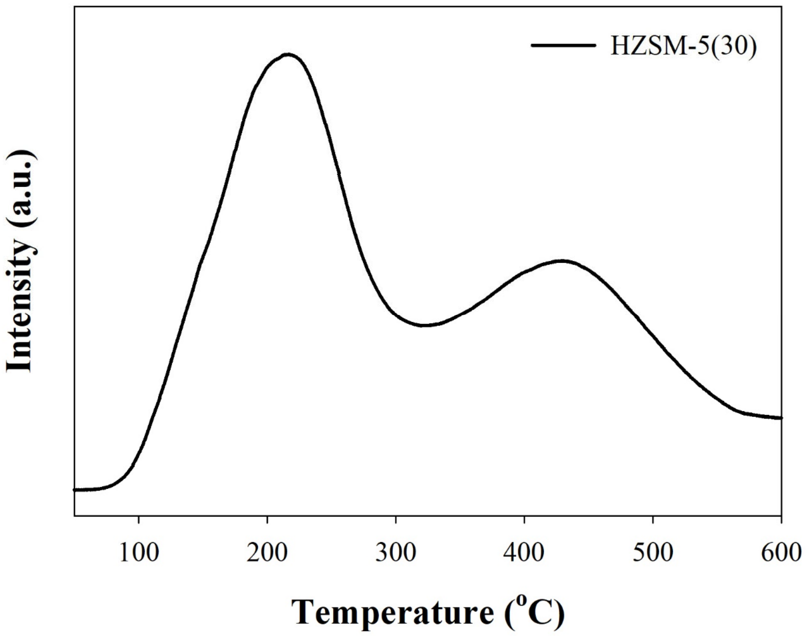

Figure S2. NH_3-TPD curve of HZSM-5(30).

Table S2. The N_2 sorption results of HZSM-5(30) catalyst used in this study.

Si/Al ratio	30
BET surface area (m^2/g)	396
Pore volume (cm^3/g)	0.25
Pore diameter (nm)	0.5

Section 3. Results and Discussion

Table S3. MS peak area of evolved chemicals under the pyrolysis reaction of CPCs.

Peak No.	Compound	MS Peak Area (×10^{-7})			
		Site A	Site B	Site C	Site D
1	1-Hexene	14.5±3.3	37.8±2.3	23.9±7.3	10.4±2.6
2	Benzene	8.3±0.2	8.8±1.7	13.7±2.1	6.3±1.6
3	1-Heptene	9.6±2.5	24.5±0.6	14.1±3.1	5.8±1.6
4	1-Heptane	3.1±1.2	13.9±0.1	5.8±1.8	2.6±0.5
5	Toluene	15.4±1.6	4.6±0.3	7.3±0.5	12.8±1.6
6	1-Octene	8.4±2.2	23.8±0.0	14.3±0.9	6.7±1.5
7	n-Octane	3.7±0.8	13.1±0.1	6.3±0.6	3.2±0.9
8	2,4-Dimethyl-1-heptene	13.6±4.2	13.7±4.1	38.1±3.7	14.4±5.4
9	Ethylbenzene	5.1±0.2	-	-	5.9±2.7
10	Styrene	109.6±0.0	28.2±0.4	19.3±1.0	210.9±1.4
11	n-Nonane	2.7±1.2	8.5±1.1	4.3±0.3	3.9±0.7
12	Methylstryene	13.9±0.5	-	-	15.3±2.0
13	Cyanobenzene	-	9.6±1.1	5.9±0.4	-
14	1-Decene	14.4±2.6	37.8±0.2	22.2±1.0	20.1±3.1
15	n-Decane	3.6±0.5	8.8±0.1	8.2±0.1	4.3±0.7
16	Dipentene	23.2±0.2	-	-	-
17	2,4,6-Trimethyl-1-nonene	1.8±0.7	1.8±0.1	5.1±0.4	3.0±1.0
18	1,10-Undecadiene	4.0±0.7	7.8±0.3	6.6±0.3	5.1±1.0
19	1-Undecene	12.3±2.2	30.9±0.5	18.0±0.6	16.7±2.6
20	n-Undecane	3.2±0.5	11.2±0.3	5.8±0.2	5.4±0.7
21	1,11-Dodecadiene	3.2±0.6	11.1±0.4	10.2±0.4	6.0±0.1
22	1-Decene	10.9±1.9	28.1±2.0	23.9±0.6	16.0±2.9
23	n-Dodecane	2.7±0.5	11.8±0.6	11.9±0.2	5.2±0.6
24	Benzoic acid	-	-	33.8±2.2	-
25	Caprolactam	-	13.0±1.0	18.6±1.0	-
26	1,12-Tridecadiene, 1-Tridecene, n-Tridecane	17.9±3.3	45.0±1.3	37.8±3.0	23.4±2.1
27	2,4,6,8-Tetramethyl-1-undecene	5.0±1.9	3.6±0.9	9.5±0.4	5.9±1.0
28	1,13-Tetradecadiene, 1-Tetradecene, n-Tetradecane	22.3±4.9	56.7±3.3	33.6±0.9	27.8±3.7
29	1,14-Pentadecadiene, 1-Pentadecene, n-Pentadecane	20.4±3.6	55.7±0.5	31.2±0.6	26.0±2.7
30	Levoglucosan	-	-	41.2±0.7	-
31	1,15-Hexadecadiene, 1-Hexadecene, n-Hexadecane	18.7±4.7	56.0±0.8	33.3±2.9	29.5±3.2
32	Terephthalic acid	-	-	7.4±0.4	-
33	1,16-Heptadecadiene, 1-Heptadecene, n-Heptadecane	16.0±5.2	55.3±0.9	29.0±1.5	27.4±4.6

34	3-Butene-1,3-diyldibenzene	-	-	-	19.3±4.9
35	1,17-Octadecadiene, 1-Octadecene, n-Octadecane	16.8±4.5	59.8±0.2	31.0±0.0	27.7±1.3
36	1,18-Nonadecadiene, 1-Nonadecene, n-Nonadecane	17.1±4.8	57.1±0.3	36.2±1.6	28.4±2.4
37	1,19-Eicosadiene, 1-Eicosene, n-Eicosane	18.2±6.0	59.6±1.9	33.4±1.2	29.9±3.8
38	1-Heneicosene	15.5±2.6	62.7±2.7	31.1±2.3	23.0±4.1
39	1-Docosene	19.4±3.4	71.6±3.6	34.9±1.7	20.8±6.5
40	1-Tricosene	16.7±1.9	72.6±4.9	33.6±2.9	22.4±2.4
41	1-Tetracosene	17.1±3.2	69.5±0.5	34.9±2.9	24.5±3.2
42	5-Hexene-1,3,5-triyltribenzene	11.5±0.7	-	5.5±2.0	38.1±16.4
43	1-Pentacosene	16.0±4.0	71.1±0.3	31.2±1.6	22.1±3.6
44	Bis(2-ethylhexyl) phthalate	44.6±0.4	-	-	-
45	1-Hexacosene	19.0±3.3	73.3±1.8	33.6±0.6	26.9±3.6
46	1-Heptacosene	17.3±2.6	76.0±1.6	40.1±2.6	25.9±3.4
47	1-Octacosene	21.5±6.7	83.8±6.4	43.1±5.0	23.0±2.6
48	1-Nonacosene	19.3±4.9	80.1±0.7	40.4±0.8	21.3±3.4
49	1-Triacontene	18.3±3.4	77.7±0.3	34.6±0.5	21.3±1.5
50	1-Hentriacontene	23.7±0.1	88.9±1.9	43.1±0.1	27.3±4.1
51	1-Dotriacontene	18.7±4.2	79.9±1.7	34.3±0.1	19.0±1.9
52	1-Tritriacontene	14.8±1.8	71.4±0.8	31.9±1.1	19.1±2.5
53	1-Tetratriacontene	14.0±2.6	85.5±11.7	30.6±0.7	18.9±2.2
54	1-Pentatriacontene	24.4±1.2	75.9±5.0	29.7±4.3	19.4±1.4
